# Abstracts presented at the Joint meeting of the 22nd Congress of the Japanese Research Society of Clinical Anatomy and the 3rd Congress of Kurume Research Society of Clinical Anatomy

**DOI:** 10.1007/s00276-020-02562-z

**Published:** 2020-09-05

**Authors:** Koh-ichi Yamaki, Keiichi Akita

**Affiliations:** 1 Department of Anatomy, Kurume University School of Medicine, Fukuoka, Japan; 2 Japanese Research Society of Clinical Anatomy (JRSCA), Tokyo, Japan

Introduction: Herewith are the abstracts of the presentations of the Joint meeting of the 22nd Congress of the Japanese Research Society of Clinical Anatomy (JRSCA) and the 3rd Congress of Kurume Research Society of Clinical Anatomy. The meeting was held at School of Medicine, Kurume University, Fukuoka, JAPAN on November 10, 2018. The theme was “Knowledge for Innovation”.

## Session 1: cardiovascular system

### Macroscopic and histological observation of the conduction system of the right atrium

#### Arakawa Takamitsu

**Kobe University Graduate School of Health Sciences**

Internodal conduction pathways of the human right atrium have been well reported in the clinical field with special reference to catheter ablation to prevent the atrial flutter. Nevertheless, detailed gross anatomy and histology of this pathway are not well documented. The conduction system can be stained by Periodic acid-Schiff (PAS) stain. 12 human cadavers obtained by Kobe University School of Medicine were examined by gross anatomical dissection and histological observation by H-E and PAS stains. Some myocardial fibers in the human right atrium could be seen as slightly whitened fibers upon macroscopic investigation. These whitened myocardial fibers could be detected in the anterior, middle and posterior intermodal tract. A part of the posterior intermodal tract was stained strongly by PAS stain. Some middle intermodal pathway could be observed as unwhitened myocardial fibers. The morphological features of these unwhitened myocardial fibers are similar to the working myocardium. These variations of the intermodal conduction pathways might be related to the pathological condition of the human right atrium.

### Investigation of variations at the sapheno-femoral junction and the sapheno-popliteal junction with plain CT

#### Sakurai Kanako, Hiromatsu Shinichi, Anegawa Tomoyuki, Kanamoto Ryo, Yoshida Shohei, Shintani Yusuke, Otsuka Hiroyuki, Tobinaga Satoru, Onitsuka Seiji, Tanaka Hiroyuki

**Department of Surgery, Kurume University, Kurume-shi, Fukuoka**

Introduction: Recently, the treatment for varicose veins (VV) is changing to minimally invasive therapy such as endovenous laser ablation (EVLA) from open surgery such as stripping and high ligation. In our department, we performed EVLA for all cases except ones with a history of deep vein thrombosis (DVT). The complications in EVLA include arterio-venous fistula and nerve injury around the sapheno-femoral junction (SFJ) and the sapheno-popliteal junction (SPJ). To prevent these complications, we should recognize the anatomy of the SFJ and the SPJ, and review the ablation area and the manner of tumescent local anesthesia (TLA). Here, we report the variations of SFJ and SPJ on preoperative plain CT.

Materials and methods: We targeted 245 limbs of 209 patients with EVLA, between September 2013 and August 2018. VV in great saphenous vein (GSV) were present in 220 limbs. VV in small saphenous vein (SSV) were present in 33 limbs. Both were present in 8 limbs.

Results: Variations of the SFJ appeared in 16 limbs (7.27%). The type of great saphenous vein crossing in front of the common femoral artery was found in 1 limb. Crossing in front of the superficial femoral artery was found in 2 limbs. Ascending in front of the superficial femoral artery was found in 13 limbs. SPJ showed no variations. After all EVLA there were no critical complications.

Conclusion: By recognizing the detailed anatomy of SFJ and SPJ before EVLA, we can prevent complications. And plain CT is useful for preoperative evaluation of the anatomy.

### Clinico-anatomical significance of the perioperative detection of an exceedingly rare superficial radial artery using MillSuss

#### Uchino Tetsuya^1^, Miura Masahiro^2^

^**1**^**Department of Anesthesiology, Faculty of Medicine, Oita University, Oita**; ^**2**^**Department of Human Anatomy, Faculty of Medicine, Oita University, Oita**

Introduction: A rare abnormal course of the radial artery, superficial radial artery (SRA), should be carefully considered during a radial artery puncture. However, the SRA is not commonly found during the preoperative physical examination and it may cause difficulty in the performance of arterial puncture and result in complications.

Materials and methods: A total of 2315 patients who underwent radial artery catheter placement was retrospectively examined. In addition, a clinical study on 200 patients with planned arterial catheter placement was conducted, and the course of the radial artery was preoperatively examined using a vascular visualization device with near-infrared light, namely MillSuss (Cardinal Health, Tokyo, Japan). The detection rate of the SRA and the success rate of the first catheter placement in the radial artery were evaluated.

Results: The SRAs were found in two patients, three arms (0.06%), during the normal preoperative medical examination. The SRAs were discovered when the radial artery was punctured in six patients, nine arms (0.19%). Only one case had a successful radial artery catheter placement during the first attempt. However, in radial arteries preoperatively examined with MillSuss, the SRAs were found in four patients, six arms (1.5%). The arterial catheter was successfully placed in all cases during the first attempt.

Conclusion: MillSuss improves the detection rate of the SRAs before the arterial puncture and the success rate of catheter placement during the first attempt because it can easily visualize and find the SRAs during the preoperative examination.

### Revisiting the anatomy of the atrioventricular node artery

#### Kawashima Tomokazu, Sato Fumi

**Department of Anatomy, School of Medicine, Toho University, Tokyo**

Introduction: Avoiding damage to the atrioventricular node artery as well as the node itself, has been one of the central issues in catheter intervention for atrioventricular nodal reentrant tachycardia (AVNRT) and valvular surgery. However, no imaging modalities can visualize the atrioventricular node itself and this limitation makes it hard to evaluate its arterial supply.

Materials and methods: One hundred and twenty embalmed human hearts were examined for this study using the gross anatomical, histological, and imaging methods.

Results and conclusion: Our visualized data showed multiple origins and courses of the atrioventricular node artery within the inferior pyramidal space. These data, indicating that the atrioventricular node artery is the only distribution-confirmed artery entering the compact node and/or its nodal extensions, clarified its accurate anatomical information and provided the gold standard for the origin, course, and distribution of the atrioventricular node artery.

### Clinical anatomical examination of the meningeal-extravascular fluid pathways related to physiological cerebrospinal fluid leakage: Scanning electron microscopic analysis

#### Miura Masahiro^1^, Miura Mako^1^, Uchino Tetsuya^2^

^**1**^**Department of Human Anatomy, Oita University Faculty of Medicine, Oita;** ^**2**^**Department of Anesthesiology, Oita University Faculty of Medicine, Oita**

Meningeal-extravascular fluid pathways (MEVFPs) are thought to be involved in the physiological mechanisms by which cerebrospinal fluid (CSF) passes through the meningeal barrier and is absorbed by the meningeal lymph vessels. In this study, we aimed to elucidate the structural features of the MEVFPs present in the human brain and spinal dura mater. In this investigation, we used meningeal-nerve root samples removed from two adult cadavers during dissection. Scanning electron microscopy (SEM) with a 2 N-NaOH maceration method was applied to demonstrate the arrangement of the collagen fibrillar network of the specific MEVFPs into dura mater. The treatment with NaOH facilitated the intercellular separation of the specimen and completely removed reticular fibers around the nerve root foramens of dura mater. In the SEM observation of sagittal sinus and periosteal arachnoid membranes, formation of small pores (mean size, 8 μm) and trabecula (like a reticular network) was commonly observed in each spinal segment. On the contrary, we observed the development of macula cribriformis (8 to 60 μm) around the neural foramen on the inner surface of C5 to C8 and L2 to S3 of spinal dura mater. This study suggests that CSF leaks quickly from the subarachnoid space (SAS) into the nerve root sheath via MEVFPs. The trabecula developing into the nerve root arachnoid membrane was thought to be a structural change that actively leads to leakage of CSF out to the intrathecal space.

## Session 2: alimentary system

### Surgical anatomy in right-sided round ligament using a 3D CT fusion image

#### Sakai Hisamune, Goto Yuichi, Akagi Yoshito, Tanaka Hiroyuki, Okuda Koji

**Department of Surgery, Kurume University School of Medicine**

Background: In the right-sided round ligament (RSRL), the anterior portal ramification is proposed to be divided into ventral and dorsal branches by the right umbilical portion (RUP), just as in the normal liver. Although the portal and venous anatomy of the liver with RSRL has already been established, the biliary and hepatic arterial anatomy of such livers has not been described in most of the studies.

Methods: RSRL was detected in 12 patients between 2006 and 2018 in our department. These 12 patients were assessed for preoperative evaluation of the hepatic vasculature and biliary system using 3D CT fusion image.

Results: Portal vein ramification was independent as the right posterior type in 7 and bifurcation type 5 patients. The right anterior portal vein bifurcated into the right ventral and dorsal portal vein in all patients. Hepatic arterial ramification was bifurcation type in 11 and replaced left hepatic artery type in one patient. The right anterior hepatic artery bifurcated into the right ventral and dorsal hepatic artery in all patients. In the course of the bile duct to the right umbilical portion, supraportal type in was found in 7 and infraportal type in 5 patients. A total type was observed in 1 patient of the infraportal type patients. In this type, independent branches from the entire left and right hemiliver were drained by a single biliary tract. The right anterior ventral hepatic duct and right anterior dorsal hepatic duct drained into the right hepatic duct or the common bile duct in all patients.

Conclusions: In the right-sided round ligament, we proposed that the anterior sector is also longitudinally divided into ventral and dorsal segments by the right umbilical portion based on the biliary and hepatic arterial findings.

### The course of replaced common hepatic artery from the superior mesenteric artery in relation to the pancreas

#### Sakai Hisamune, Goto Yuichi, Akagi Yoshito, Tanaka Hiroyuki, Okuda koji

**Department of Surgery, Kurume University School of Medicine**

Background: Although the incidence of the replaced common hepatic artery from the superior mesenteric artery (RCHA) as the hepatomesenteric trunk (HMT) has been reported in the literature to range from 0.4 to 4.5%, the course of this anomaly to the pancreas has not been described in most of the studies.

Methods: RCHA was detected in 13 patients between 2010 and 2018 in our department. The routine preoperative 3-dimensional computed tomography of the 13 patients were analyzed, and the courses of the anomalous RCHA in relation to the pancreas were classified.

Results: The following three patterns were identified in the course of RCHA: Type Ia (*n* = 8), RCHA from the HMT running in the dorsal aspect of the head of the pancreas. Type Ib (*n* = 3), RCHA from the HMT running through the pancreatic parenchyma. Type II (*n* = 2), RCHA from the pancreatic arcade running along the ventral surface of the pancreas in a similar course as the gastroduodenal artery. Pancreatoduodenectomy was performed for the middle part of the bile duct cancer with RCHA running through the pancreatic parenchyma. The pancreas head was transected and RCHA was carefully isolated without injury. RCHA ran between the ventral pancreas and the dorsal pancreas.

Conclusion: The surgeon must recognize not only vascular anomalies but also the course of the vessels prior to pancreatic resection. We proposed a classification of the RCHA that divided into three types from the embryological development of the hepatic artery and the fusion of the ventral and dorsal pancreas.

### The hepatic capsular artery and its clinical utility

#### Ibukuro Kenji^1,2^, Nasu hisayo^2^, Yamaguchi kumiko^2^, Akita Keiichi^2^

^**1**^**Department of Radiology, Nihon University School of Medicine, Tokyo; **^**2**^**Department of Clinical Anatomy, Tokyo Medical and Dental University, Tokyo**

Introduction: To evaluate the hepatic capsular arteries and their clinical utility.

Methods: (1) Cadaver: Barium sulfate was injected into the hepatic artery, cystic artery (CA), right inferior phrenic artery (RIPA), and falciform ligament artery, then the hepatic capsular arteries were evaluated. (2) Clinical cases: We reviewed the abdominal angiography to find the cases in whom the hepatic capsular arteries were related. We also reviewed the contrast-enhanced CT scans in patients with hepatic artery occlusion to find collateral vessels around the liver.

Results: (1) There are two types of hepatic capsular arteries, the short and long types. The former type distributed branches around 2–3 cm, and the latter type distributed the branches around several cm. The CA demonstrated the capsular arteries at the gallbladder fossa. The capsular arteries at the posterior aspect and S1 were demonstrated by the RIPA. The falciform ligament artery branched the capsular arteries at the anterior aspect of S4. (2) Case 1: A 50 s male with hepatocellular carcinoma. When the hepatic artery was embolized by Lipiodol, the CA was also demonstrated via the capsular artery at the gallbladder fossa. Case 2: A 40 s female with vasculitis. The proper hepatic artery was occluded. The contrast-enhanced CT scan showed the collateral vessels at the posterior aspect of the liver via the RIPA and at the falciform ligament via the internal thoracic artery.

Conclusion: The communications between the hepatic artery and the extra-hepatic arteries were developed not directly but through the hepatic capsular arteries.

### Atherosclerosis of the right posterior hepatic artery in a patient with hilar cholangiocarcinoma undergoing left trisectionectomy: a case report of a therapeutic pitfall

#### Goto Yuichi, Sakai Hisamune, Okuda Koji

**Division of Hepatobiliary and Pancreatic Surgery, Department of Surgery, Kurume University School of Medicine, Kurume, Japan**

Background: We encountered a rare case of benign stricture of the right posterior hepatic artery (RPHA) caused by atherosclerosis.

Case: A 75-year-old man was referred to us for the investigation of serum hepatobiliary enzyme elevation. The patient had a history of hypertension, diabetes mellitus, and an operative history of coronary artery bypass grafting. Endoscopic retrograde cholangiography revealed strictures of the bilateral hepatic ducts with the involvement of the right anterior and posterior bile ducts. Adenocarcinoma was evident by cytology. We diagnosed these findings as hilar cholangiocarcinoma and planned left trisectionectomy including bile duct reconstruction. Although the tumor and RPHA were not adjacent, preoperative CT revealed a stricture of the RPHA that was 5.6 mm in length. We suspected that atherosclerosis caused the stricture. Digital subtraction angiography was performed and intravascular ultrasonography showed stricture of the RPHA accompanied by thick plaques in the arterial wall. A bare-metal stent was placed in the RPHA and then left trisectionectomy was performed. Since this patient developed bile leakage postoperatively, the bile leakage was controlled, and the patient was discharged three months after surgery. Unfortunately, four months after hepatectomy, he was re-hospitalized with multiple pyogenic liver abscesses. We performed intensive multimodal treatment for the liver abscesses and stabilized the disease, however, he eventually died due to liver failure 14 months after surgery.

Conclusion: There is no previous literature on atherosclerosis of the RPHA, which was evident preoperatively. We speculate that existing chronic microscopic injury of the peribiliary plexus might have caused the liver abscesses.

### Muscular architecture of the abdominal part of the esophagus and stomach

#### Hur, Mi-Sun

**Department of Anatomy, Catholic Kwandong University College of Medicine, Gangwon-do, Korea**

The aim of this study was to clarify the muscular architecture of the abdominal part of the esophagus and stomach. Anatomical examination was performed on 60 stomachs and esophagi from embalmed Korean adult cadavers. The anterior and posterior longitudinal fibers of the esophagus descended and blended serially with the circular fibers of the stomach adjacent to the upper lesser curvature in all specimens (100%). In the abdominal part of the esophagus, the circular fibers of the esophagus on the right side descended obliquely toward the left side to become the circular fibers of the stomach. And the circular fibers of the esophagus on the left side descended obliquely toward the right side to become the oblique fibers in all specimens. The circular fibers and oblique fibers of the stomach which originated from the abdominal part of the esophagus crossed each other in the cardial orifice in all specimens (100%). The arrangement of the circular fibers of the stomach on the posterior surface differed from that on the anterior surface in all specimens (100%). On the posterior surface of the upper stomach, the circular fibers and longitudinal fibers on the greater curvature were merged and ran parallel toward the fundus. On the posterior surface of the body of the stomach, the circular fibers ran straight between the lesser and greater curvatures. On the anterior surface of the stomach, the circular fibers encircled the stomach and lay beneath the longitudinal fibers on the lesser and greater curvatures. In the body of the stomach, the circular fibers were divided into several fibers before encircling the greater curvature. On the internal surface of the stomach, the oblique fibers lay on the abdominal part of the esophagus, cardial part, body, and fundus of the stomach. Near the greater curvature, the ends of most oblique fibers were connected to the circular fibers. Near the lesser curvature, the most upper oblique fibers were connected to the circular fibers, serially. From the body to fundus of the stomach, the direction of the oblique fibers fanned out toward the fundus. Then the oblique fibers encircled the projected point of the fundus. These anatomic data regarding muscular architecture of the esophagus and stomach will contribute toward the understanding of form and motility of the stomach under various conditions.

## Special lecture

### Current and future trends/developments in clinical anatomy education/research in the United States

#### Tubbs R. Shane

**Seattle Science Foundation, Seattle, Washington, USA**

Introduction: The time devoted to anatomical training, both didactic and dissection, to medical trainees has dwindled over the past decades. This has resulted in medical graduates with a loose foundation during their postgraduate training and professional careers. Additionally, fewer universities are training teachers and researchers in anatomy, especially its clinical applications.

Methods: Herein, the author will discuss such decreases in anatomical education and research as currently seen in the United States.

Results: The lack of clinically relevant anatomical training programs, decreased emphasis of anatomical training for undergraduate medical students, lack of NIH funding for clinically relevant anatomical studies, and an overall sense that students will absorb what anatomy they need to know during their training and professional careers are just some of the obstacles adding to the already dire situation of anatomical education and research in the United States.

Conclusion: The current trends of de-emphasizing anatomy in medical education seem to have escalated and the number of classically trained anatomists who are teaching undergraduate medical trainees is at an all-time low. Additionally, fewer researchers are focused on clinically related research. The former problem may be too far gone to remedy but the later can be bolstered with emphasis to students and researchers alike regarding the utility of clinically relevant anatomical research.

## Session 3: locomotor system

### An anatomic study on the origin of the long head of the triceps brachii

#### Nasu Hisayo^1^, Nimura Akimoto^2^, Akita Keiichi^1^

^**1**^**Department of Clinical Anatomy, Graduate School of Medical and Dental Sciences Tokyo Medical and Dental University, Tokyo;** ^**2**^**Department of Functional Joint Anatomy, Graduate School of Medical and Dental Sciences Tokyo Medical and Dental University, Tokyo**

Introduction: Causes of posterior shoulder instability are considered to be injuries of the posterior parts of the joint capsule, the labrum or the inferior glenohumeral ligament. The origin of the long head of the triceps brachii (LHT) is also located at the posteroinferior region of the glenohumeral joint. However, the relationship between the LHT and the surrounding structures has not been clarified. Therefore, the purpose of the current study was to clarify the detailed morphology of the origin of the LHT.

Materials and methods: A total of 64 specimens from 36 cadavers (11 males and 25 females) were used. After dissecting the origin of the LHT in 54 specimens of 27 cadavers, the width of the origin of the LHT was measured with a caliper. The origin of the LHT was also investigated histologically in 18 specimens. Sections were analyzed with Masson’s trichrome staining and Safranin O staining.

Results: First, the origin of the LHT had a developed fibrocartilage. Second, descending fibers on the dorsal surface of the scapula mixed with the LHT. The width of the origin of the LHT on the dorsal surface of the scapula was 31.23 mm. Third, the LHT was fused with the glenohumeral joint capsule and it was directly attached to the glenoid labrum. Fourth, we showed that the LHT formed a bowl-shaped structure at a simulated position.

Conclusions: The LHT could affect the glenohumeral joint capsule or the glenoid labrum because of their connections to each other and could be indirectly associated with posterior shoulder instability.

### Investigation of the fifth muscle head of the quadriceps femoris

#### Ogami–Takamura Keiko^1,2^, Saiki Kazunobu^1^, Okamoto Keishi^1^, Tsurumoto Toshiyuki^1^

^**1**^**Department of Macroscopic Anatomy, Nagasaki University School of Medicine, Nagasaki;** ^**2**^**Department of Anesthesiology, Nagasaki University Hospital, Nagasaki**

Introduction: The quadriceps femoris is commonly recognized to consist of four muscle heads: rectus femoris, vastus lateralis (VL), vastus intermedius (VI), and vastus medialis. However, Grob et al. (Clin Anat 29:256-263, 2016) reported a new muscle head between VL and VI, named tensor of VI (TVI). We investigated the existence of TVI in Japanese cadavers and studied its morphology.

Materials and methods: Thirty-one sides of 16 formalin-preserved Japanese cadavers were investigated. TVI was classified into four types according to the classification of Grob et al. (Clinical Anatomy 29:256–263, 2016). We measured the length of the muscle bellies and aponeuroses. Furthermore, the nerves and arteries for TVI were identified. We evaluated the presence of TVI using postmortem CT images.

Results: At least one TVI was identified in each side. Two TVI heads were identified in one side. Overall, VL type was 38%, VI type was 25%, Independent type was 9%, Common type was 28%. The length of the muscle belly was 105.5 ± 35.0, and the tendon was 213.7 ± 35.7 [mean (mm) ± SD]. The nerves to TVI muscle originated from the femoral nerve directly (22 sides), from the branches for VI muscles (5 sides), from the branches for VL muscle (5 sides). Each dominant artery originated from the lateral circumflex femoral artery. Depending on the case, the TVI could be confirmed in the postmortem CT images.

Conclusion: We identified TVI in each lower extremity of Japanese cadavers. It was possible to identify TVI in the postmortem CT. It is necessary to examine the clinical significance of this new muscle.

### New origin of the vastus lateralis—the frontal part of the capsule of the hip joint

#### Yoshida Daichi^1,2,^,Tabira Yoko^3^, Saga Tsuyoshi^3^, Nooma Kunimitu^1,4,^ Watanabe Koichi^3,^ Yamaki Koh-ichi^3^

^**1**^**Master’s course of Kurume University Graduate School of Medicine, Kurume, Fukuoka** ^**2**^**Medical Association Housenkai Maruyama Hospital, Ogoori, 838-0113, Fukuoka** ^**3**^**Department of Anatomy, Kurume University School of Medicine, Kurume, Fukuoka** ^**4**^**Miyuki Hospital, Kumamoto**

The origin of the vastus lateralis (VL) is generally considered to be the base of the great trochanter, the gluteal tuberosity, and the lateral lip of linea aspera. However, we found a case which had a new origin of the VL, the capsule of the hip joint. The purpose of this study was to clarify this new origin in gross anatomical and histological examinations. We investigated 20 preserved cadavers in anatomical dissection courses. Prior to the dissection, we measured the bilateral, femoral length and circumference. After removing the skin, we dissected the left hip joint and examined whether VL had the origin on the capsule. The cases were classified into two groups: the cases which had the origin on the capsule (Ct) and the cases which did not have the origin on the capsule (NCt). We measured the outer circumference and the area of the origin of each muscle of quadriceps femoris and confirmed the relationship statistically by Wilcoxon’s rank-sum test for these data as comparative factors. Ct was found in 4 of the 20 limbs (20%). The circumference of the thigh in the Ct was significantly smaller than in the NCt (*P *< 0.01) and the area on the capsule of the rectus femoris in Ct was significantly smaller than NCt (*P* < 0.01). We presume that the cranial extension of the VL to the capsule supplements the narrow origin on the capsule of the rectus femoris in Ct.

### New findings in a female pelvis obtained from a Thiel-fixed body taken with an 8 K video camera

#### Kato Tomoyasu^1^, Kato Mayumi^1^, MURO Satoru^2^, Yamashita Hiromasa^3^, Tanioka Kenkichi ^3^, Chiba Toshio^3^, Akita Keiichi^2^

^**1**^** Department of Gynecology, National Cancer Center Hospital, Tokyo;** ^**2**^** Department of Clinical Anatomy, Tokyo Medical and Dental University, Tokyo;** ^**3**^** Kairos Co., Ltd., Tokyo**

Introduction: An 8 K video camera provides high definition and high-resolution images. New findings of the female pelvis regarding the courses of vessels and autonomic nerves were obtained using the 8 K video camera and investigated for application to nerve-sparing radical hysterectomy.

Materials and methods: One female pelvis of cadaver fixed by the Thiel method was studied at Tokyo Medical and Dental University. The dissection was performed in the same procedure as radical hysterectomy.

Results: The findings not obtained in previous studies are listed.

- A blood vessel on the surface of the hypogastric nerve was found.

- The ureterohypogastric fascia wrapped the ureter around the intersection of the uterine artery where the small vessels surrounding the ureter were observed.

- The branches toward the uterus of the autonomic nerves were clearly shown when the visceral stump of the cardinal ligaments was mobilized ventrally.

- The autonomic nerves from the ureterohypogastric fascia distributed to the bladder over the ureter were observed when developing the anterior layer of the vesicouterine ligament.

- The autonomic nerves running on the lateral surface of the ureterohypogastric fascia were seen, after cutting the posterior layer of the vesicouterine ligament.

Conclusion: To reveal the small vessels surrounding the ureter by an 8 K video camera may lead to a decrease in the occurrence of adverse events, such as damage of the ureter. Recognition of the distribution of autonomic nerves to the bladder may provide a landmark to determine the cutting line of the rectovaginal ligament.

### Anatomic and Imaging Diagnostic Analyses of Pelvic Floor (PF) Aging

#### Okuda Itsuko^1,2^, Yoshioka Naoki^1^, Akita Keiichi^2^, Ohota Hiroaki^3^

^**1**^** Department of Diagnostic Radiology, International University of Health and Welfare (IUHW), Mita Hospital Tokyo**; ^**2**^** Department of Clinical Anatomy, Tokyo Medical and Dental University (TMDU), Tokyo;** ^**3**^** Women’s Medical Center, Sanno Medical Center, Tokyo**

Introduction: In a super-aging society, the number of patients suffering from pelvic organ prolapse (POP) is increasing and many women are becoming more concerned about it. The pelvic floor (PF) fragility, attributable to PF aging, is regarded as one of the factors contributing to POP, as the PF drops with aging. However, the process of change with the PF descensus is not fully known. Therefore, the PF configurations using MRI were analyzed based on anatomy and then assessed in association to aging.

Materials and methods: Anatomic studies were done in the department of clinical anatomy at TMDU. MRI examinations using a 1.5-T superconducting MRI apparatus were performed in the IUHW-related hospitals. The pelvic MR images of 74 healthy women from 20 to 91 years of age were enrolled. Based on the anatomical findings, MR imaging findings were analyzed. The anatomic structures constituting the PF were identified, and their changes were evaluated according to the age of the subjects.

Results: MRI clearly revealed the structures of the PF involved in aging. In particular, the morphological changes of the PF could be easily distinguished in the coronary images of the T2-weighted images. PF configurations were different between the young and the elderly, and the PF had drooped with aging.

Conclusion: To diagnose PF aging, it is important to understand PF anatomy and the features of physiological changes associated with aging. MRI could depict the anatomic structures related to PF aging. Our methods of analysis could provide useful information to the gynecology and urology involved in the diagnoses and treatments of POP.

## Session 4: head and neck

### Anatomical features of the mentalis muscle and zygomaticus minor muscle

#### Hur Mi-Sun

**Department of Anatomy, Catholic Kwandong University College of Medicine, Gangwon-do, Korea**

The aim of this study was to clarify the morphological patterns and anatomical variations of the mentalis muscle (MT) and zygomaticus minor muscle (Zmi) in relation to the surrounding muscles. MTs and Zmis were examined in 40 specimens of embalmed adult Korean cadavers.

The medial fibers of both MTs descended anteromedially and crossed each other forming a dome-shaped chin prominence in all specimens (40/40 specimens, 100%). They then attached to the chin skin of the other side. The lateral fibers of the MT were intermingled with the depressor labii inferioris muscle to attach to the skin of the same side. The upper fibers of the MT were short and ran horizontally, whereas the lower fibers were long and descended inferomedially or vertically. The upper fibers of the MT were intermingled with the inferior margin of the orbicularis oris muscle (OOr) in all specimens (40/40 specimens, 100%). The medial fibers of the MT crossed each other horizontally, just below the central portion of the OOr inferioris. These two muscle fibers were arranged in continuous horizontal layers. The originating muscle fibers of the incisivus labii inferioris muscle were intermingled with the upper lateral MT in all specimens. Some of the ILI fibers extended inferomedially to the middle or lower portion of the MT.

The Zmi was present in all specimens (32/32 specimens, 100%). In 96.9% (31/32 specimens), the Zmi that inserted into the upper lip was formed by muscle fibers that arose from the zygomatic bone and muscle fibers that extended from the orbicularis oculi muscle (OOc). In 43.8% (14/32 specimens), some fibers of the Zmi that arose from the zygomatic bone blended with the inferior margin of the OOc, while the other fibers inserted into the upper lip. After the Zmi fibers blended with the inferior margin of the OOc, these fibers constituted the inferior and medial margins of the OOc. These fibers were then attached to the medial palpebral ligament, the maxilla, the levator labii superioris alaeque nasi muscle, and the depressor supercilii muscle.

The obtained data will be helpful for understanding their connected movements and in kinematics and electromyographic analyses, therapies involving injections of botulinum toxin type A, and various types of facial surgery.

### Clinico-anatomical study on the relationship between the location of the mandibular canal and postoperative paralysis in sagittal split ramus osteotomy

#### Shinozaki Katsumi^1^, Todoroki Keita^1^, KIKUTA Syogo^1,2^, Iwanaga Jyo^1,2^, Kusukawa Jingo^1^

^**1**^**Dental and Oral Medical Center, Kurume University of Medicine, Fukuoka;** ^**2**^**Seattle Science Foundation, Washington, USA**

Introduction: Sagittal split ramus osteotomy (SSRO) is the most common operation for mandibular prognathism, but the occurrence of neurosensory disturbances of the inferior alveolar nerve (IAN) is a frequent complication. We evaluated the anatomical location of the IAN canal (IAN Can) using cone-beam computed tomography (CBCT) and examined the relationship between an appearance of postoperative neurosensory disturbances and the recovery.

Materials and methods: CBCT (slice 1.0 mm in width, 0.5 mm interval) images were taken from 106 patients (206 sides) with jaw deformity who underwent SSRO. The minimum distance from the buccal aspect of IAN Can to the inner aspect of the outer cortex of the mandibular ramus (Dcc) was measured. According to the positional relation between IAN Can and the outer cortex of the mandibular ramus, the cases were classified into 3 types (separated, contact and fused). Intraoperative exposure of the IAN bundle in the operation field was also observed. Furthermore, the relationship of Dcc with occurrence and improvement of the postoperative paralysis of IAN was evaluated. Paresthesia was subjectively evaluated immediately and 6 months after the operation.

Results: The separated, contact and fused types were observed 151 sides (73.3%), 45 sides (21.3%), and 10 sides (4.9%), respectively. Even in separated type, Dcc less than 0.8 mm increased the risk of IAN exposure. Patients with Dcc of less than 1.7 mm had a significantly increased risk of incidence of neurosensory disturbance. The recovery from paralysis after 6 months was significantly worse in the IAN exposure group.

Conclusion: The location of the IAN Can significantly influences the incidence and recovery of the IAN paralysis in SSRO operation.

### Anatomical and Clinical Study of the Sac which exists below the Opening of the Nasolacrimal Duct

#### Tanaka Kengoh^1^, Saga Tsuyoshi^2^, Nooma Kunimitsu^3^

^**1**^**Tanaka Ophthalmology Clinic, Yamaga City, Kumamoto;** ^**2**^**Department of Anatomy, Kurume University School of Medicine, Fukuoka;** ^**3**^**Master’s course of Kurume University Graduate School of Medicine, Fukuoka**

The Sac is a small pocket-like structure existing below the opening of the nasolacrimal duct. The inferior nasal meatus region of the human nasolacrimal duct consists of a duct and an opening that may have some attachments, including, in part, a large part of the duct (ampulla), a thin membranous structure above the opening, and a hollow area and sac below the opening. The sac and opening’s structure are very important parts of the nasal cavity during surgery (e.g., to treat epiphora). In this study, we focused on the region below the opening of the nasolacrimal duct in 50 adult Japanese cadavers at a gross anatomy laboratory. Our results showed that the shape of the opening could be classified into six types: wide opening (17%); small opening (15%); pinhole opening (14%); fissure (32%) and pseudo-obstruction (22%). In this region below the opening, 85% of cases had a hollow area that continued from the opening, and 67% had a small sac. We prepared thin sections of the opening and sac, stained them with H&E, and recorded detailed observations. Reconstructed 3D models were prepared using Neurolucida software. The size of the sac was about 1.4 mm depth, (Max. 3.2 mm, Min. 0.2 mm). The epithelium of the sac consisted of pseudostratified ciliated epithelia cells and several goblet cells like the nasal epithelium. We concluded that this knowledge is the basis for a useful approach to the nasolacrimal duct and its opening, facilitating successful outcomes for surgical interventions in the nasal cavity.

## Session 5: lymphoid system

### Challenge to dissect lymphatic vessels in the inguinal region of a human cadaver fixed with the saturated salt solution method

#### Omotehara Takuya, Kawata Shinichi, Shimada Kazuyuki, Ogawa Yuki, Itoh Masahiro

**Department of Anatomy, Tokyo Medical University, Tokyo**

Introduction: Anatomical knowledge of the lymphatic system in the lower extremity is important for injection of the contrast medium to visualize the thoracic duct and for understanding the cause and treatment of edema in the lower extremity. There are, however, no topographical pictures including the lymphatic systems in this region. In this study, we tried to obtain topographical information on inguinal lymphatic systems using a fixed human cadaver.

Materials and methods: The lymphatic system in the left inguinal region was carefully dissected in a human cadaver (male, senility) fixed with the saturated salt solution (SSS) method which can be used to attain a result that is similar to the soft condition of a fresh cadaver.

Results: Some lymph nodes were found around the saphenous hiatus, and lymphatic vessels, which were thin, yellowish, and inelastic ducts, were running between the nodes. Connections of the lymphatic vessels from the surface to the femoral canal were observed at the saphenous hiatus. The biggest lymph node was located at the lateral and caudal side of the saphenous hiatus; a lateral accessory saphenous vein and a cutaneous branch of femoral nerve were found adjacent to the node.

Conclusion: The clear network of the lymphatic system in the inguinal region was observed. A vein and nerve may run adjacent to a large lymph node which appears to be useful for injection of a contrast medium. The SSS method offers a continuous soft condition like a fresh cadaver, which is suitable for anatomical research of lymphatic systems.

### Gross anatomy of the lymphatic system in the face and neck—Distribution of the lymphatic system around the spinal dura mater and the lateral posterior wall of the pharynx

#### Kawata Shinichi^1^, Shimada Kazuyuki^1^, Omotehara Takuya^1^, Naito Michiko^2^, Ogawa Yuki^1^ Aizawa Shin^2^, Itoh Masahiro^1^

^**1**^**Department of Anatomy, Tokyo Medical University, Tokyo;** ^**2**^**Division of Anatomical Science, Department of Functional Morphology, Nihon University School of Medicine, Tokyo**

Introduction: Gross anatomical knowledge of the lymphatic vessels and lymph nodes are important information for surgical treatment of tumors at the head and neck region. We developed a new antiseptic treatment method that mainly consisted of the saturated salt solution (SSS). The fixation facilitated the identification of the lymphatic vessels and lymph nodes as distinguishable from other tissues. The purpose of this study was to determine the distribution of the lymphatic vessels and lymph nodes at the spinal dura mater and near the posterior wall of the pharynx.

Materials and methods: We used a senility cadaver. The dissection at the posterior neck was done from the surface to deep layer by gross eyes and macroscopic scope.

Results: The lymphatic vessels and lymph nodes at the posterior cervical region were separately observed in three layers. (1) Superficial layer: The lymphatic vessels at the posterior occipital region communicated with the auricle nodes. (2) Middle layer: The lymphatic vessels at the adjacent spinous processes were connected to the surface of the spinal dura mater. (3) Deep layer: The deep cervical lymph nodes were connected to the lymph nodes of the inferior posterior pharyngeal wall and to the lymph nodes at the posterior side of the esophagus.

Conclusion: In this study, the continuity of the lymphatic vessels in the surface and deep layers of the head and neck could be observed easily using the SSS method. These results are applicable to the surgical treatment of the head and neck.

(COI: none).

### Patterns of the blood supply to the mammary sentinel lymph nodes necessary for breast cancer metastasis: Clinical anatomical analysis

#### Abe Miyuki^1,2^, Miura Mako^1^, Zaitsu Sumika^1^, Uchino Tetsuya^3^, Miura Masahiro^1^

^**1**^**Department of Human Anatomy, Oita University Faculty of Medicine, Oita;** ^**2**^**Department of Thoracic and Breast Surgery, Oita University Faculty of Medicine, Oita**; ^**3**^**Department of Anesthesiology, Oita University Faculty of Medicine, Oita**

In recent years, a new mechanism of hematogeneous metastasis has been proposed in which breast cancer cells flowing into the lymph node (LN) via intracortical blood vessels cause hematogenous metastasis. In this study, we attempted anatomical and histological investigations of the morphological features and vascular patterns of the mammary sentinel lymph nodes (MSLNs) of 8 axilla from eight cadavers subjected to dissection. MSLNs and other axillary LNs were dominated by multiple branches from different origins. In all cases, fusion was recognized between MSLNs. Histological examination revealed that the development of internode septum within the fused LN was poor, and as for characteristic findings, we observed dilatation of the hilar region and an increase in the number of afferent blood vessels. Based on the patterns of blood supply to MSLNs, we suspected that developmental factors affect cortical hematogenous metastasis, based on the patterns of blood supply by different blood branches derived from different origins. According to the patterns of the blood supply to MSLNs by different blood branches derived from different origins, we suspected that developmental factors affect cortical hematogenous metastasis. On the contrary, the MSLN fusion formation was considered to be a structure susceptible to hematogegnous metastasis via high endothelial venule, due to an increase in the number of afferent lymphatic vessels and blood vessels running to or from a single LN.

### Clinical anatomical examination of popliteal lymphatic system with special reference to the popliteal lymph node metastatic pathway of malignant melanoma of the foot and ankle

#### Miura Masahiro^1^, Abe Miyuki^1,2^, Miura Mako^1^, Uchino Tetsuya^3^

^**1**^**Department of Human Anatomy, Oita University Faculty of Medicine, Oita;** ^**2**^**Department of Thoracic and Breast Surgery, Oita University Faculty of Medicine, Oita;** ^**3**^**Department of Anesthesiology, Oita University Faculty of Medicine, Oita**

Introduction: In recent years, lymph node clearance has been considered an important surgical treatment for metastatic spread of malignant melanoma of the foot and ankle to the popliteal lymph nodes (PLNs). However, little is known of the precise anatomical information required for PLNs clearance. In this study, we performed a detailed anatomical investigation of PLNs to improve the quality of life after PLN clearance.

Materials and methods: The specimens were taken from 4 dissected bodies of adult cadavers in Oita University. We examined the whole popliteal lymphatic system using the reverse-dissection method to demonstrate the communication pattern between the superficial and the deep lymphatic vessels in the popliteal fossa.

Results: In all cases, PLNs were found as three relatively small lymph nodes between the knee joint posterior capsule and the popliteal vascular sheath. The entry of the rich lymphatic vessels arising from the intercondylar area and deep lymphatic vessels accompanying the vascular sheath were observed. However, one case had entry from the subcutaneous superficial lymphatic vessel accompanying the small saphenous vein.

Conclusion: These results suggested that the PLNs were classified into parts of the deep layer lymphatic system of popliteal fossa. In the popliteal hot nodes as the metastatic pathway of foot melanoma, it was assumed to show that individual differences play an important role as sentinel lymphatic nodes of the subcutaneous superficial lymphatic system of the foot and ankle.

